# An Outbreak of Hepatic Veno-Occlusive Disease in Western Afghanistan Associated with Exposure to Wheat Flour Contaminated with Pyrrolizidine Alkaloids

**DOI:** 10.1155/2010/313280

**Published:** 2010-06-28

**Authors:** Faizullah Kakar, Zarif Akbarian, Toby Leslie, Mir Lais Mustafa, John Watson, Hans P. van Egmond, Mohammad Fahim Omar, Jawad Mofleh

**Affiliations:** ^1^Ministry of Public Health, Kabul, Afganistan; ^2^London School of Hygiene and Tropical Medicine, Keppel Street, London WC1E 7HT, UK; ^3^World Health Organisation, Geneva, Switzerland; ^4^National Institute for Public Health & the Environment, RIVM, 3720 BA Bilthoven, The Netherlands

## Abstract

Pyrrolizidine alakloids (PAs) are known to cause hepatic veno-occlusive disease (VOD). Outbreaks have occurred in Western Afghanistan since 1974, the latest in February 2008. We conducted an outbreak investigation using a case-control design. Sixty-seven cases of VOD were compared with 199 community controls. Consumption of bread was strongly associated with disease (adjusted odds ratio: 35.8 [95%CI: 7.6–168.2]). Toxic doses of PA were found in plant extracts and in samples of wheat flour taken from the study area. Compared to wheat flour there was 1000 times less PA in milk and whey and in water samples the PA content was zero. Although direct analysis was not possible, contaminated wheat flour used to make bread was the likely source of PA causing the outbreak. Eating a more varied diet including meat and fruit may be protective. Prevention and control measures will rely on community awareness and agricultural interventions to ensure safety of the food supply.

## 1. Introduction

Hepatic veno-occlusive disease (VOD), where toxic exposure causes rapid onset of massive ascites, has historically been called Gulran Disease in Afghanistan because it has consistently occurred in Gulran district of Herat Province, Western Afghanistan. While first reported as “bread poisoning” in 80 cases in South Africa in 1920 [1] and then as “camel belly” in about 1500 cases in Uzbekistan starting in 1931 and 1945 [2], the largest outbreak ever reported was from 1974 to 1976 in Gulran District affecting an estimated 7800 people with about 1600 deaths. “Gulran Disease” was attributed to consumption of bread made from wheat contaminated with seeds of a weed, locally called *charmac* [3], and hepatic veno-occlusive disease was diagnosed on liver biopsy [4]. The weed plant species was identified as *Heliotropium popovii* H. Riedl subsp. *gillianum* H. Riedl and was shown to contain pyrrolizidine alkaloids (PAs), primarily heliotrine [5]. 

Numerous PAs have also been found to cause VOD and as many as 3% of the world's flowering plants (6000 species) contain PAs [6]. PAs are not toxic* per se* but undergo metabolic oxidation by hepatic multifunction oxidases to pyrrole derivatives, which then undergo hydrolysis to strongly alkylating metabolites [7]. The dose and duration of exposure to heliotrine required to produce liver damage in humans has been previously estimated as 4–10 mg/kg per day for 3–7 weeks [8]. WHO has indicated that the lowest intake causing disease may be 1 mg total PAs per day for a 70 kg adult [9], and German regulations for herbal remedies establish a maximum oral intake of 1 microgram per day [10]. 

Without effective action in the interim, a second outbreak occurred from 1999 to 2001 with estimated 400 cases and over 100 deaths [11], and on 19 February 2008, Afghanistan's outbreak and disease surveillance system, the Disease Early Warning System (DEWS) team responded to rumors of a further outbreak of Gulran Disease in the same district. They identified 38 cases of massive ascites and four deaths which appeared to be associated with consumption of contaminated wheat flour [12]. 

The DEWS Team noted that farmers were not convinced of the cause of disease pointing out that the charmac weed is present every year throughout Gulran District, but the disease was reported from only five out of 230 villages. Furthermore, in large households, only one or two persons had the disease yet they all ate the same bread made from the same flour. In response to the outbreak, we conducted a case-control study amongst the population of Gulran District in order to better understand specific risk factors and to help guide control measures.

## 2. Methods

### 2.1. Study Area

Gulran district has an area of approximately 150 km^2^ with a population estimated at 110,000 people [13]. It consists of undulating hills with scanty vegetation serving as pasturelands. Water is scarce, salty in some places, and obtained from shallow wells and small springs. During winter the villages are snowbound and totally isolated. The inhabitants are illiterate, mostly wheat farmers who also may raise legumes, and many keep sheep and goats. Their diet consists of wheat bread and occasionally meat. In 2007-2008 the level of rain was normal and there were no reports of water shortages.

### 2.2. Selection of Cases and Controls

Active case detection was conducted, starting with cases from the District Hospital and then going house-to-house in villages of the cases. Cases were defined as having all three of the following: (1) residence in Gulran district; (2) acute appearance of massive ascites occurring in the last 6 months; and (3) no edema on face and arms. Two controls were matched by age-group and sex with each case and systematically selected, from neighboring households in the same village, and from other nearby villages which had not reported cases. One additional control was selected from within affected households, although it was not possible to match these controls for age group. Sample size for analysis included all cases identified during the active case detection.

### 2.3. Procedures and Questionnaire

All activities were conducted under the auspices of the Ministry of Public Health of Afghanistan. The case-control study was conducted as part of an outbreak investigation, and verbal consent was obtained from cases and controls prior to interview. Interviews were conducted in April and May 2008.

Cases and controls were interviewed in local language (Persian Dari) by trained Afghan surveyors using a structured questionnaire format. The questionnaire consisted of six sections: Demographic and socioeconomic details including farm size, number of animals, and water availability; clinical and treatment history; food consumption history; food and wheat preparation; knowledge of VOD; and a mortality report to ascertain if any household members had died of suspected VOD in the last six months.

Because food consumption differs considerably between seasons, cases and controls were asked about frequency of consumption of 33 food items commonly consumed in Afghanistan during a typical week in the winter and in the summer. Assuming flat-bread (naan) consumption to be ubiquitous, we asked how often bread was consumed as the only food in a meal.

Serum was collected from both cases and controls and analyzed for levels of aspartate aminotransferase (SGOT), alanine aminotransferase (SGPT), bilirubin, Hepatitis B, and Hepatitis C. 

Samples of flour, grain, and plant materials were collected where it was available (as a convenience sample) from the houses of cases and controls and stored for later analysis for PA content. As goats graze on the charmac weed, we also collected samples of goat's milk, the dried whey product called *qurut*, and water to examine if PAs are present [14]. 

### 2.4. Laboratory Methods

PA content was assessed by means of liquid chromatography/mass-spectrometry. Test portions (flour, *qurut,* and milk) were weighed and extracted with a solvent mixture of 30% water and 70% methanol, containing 5% acetic acid (flour and *qurut*) or with 100% methanol containing 0.1% acetic acid (milk). After centrifugation, an aliquot was taken from the clear supernatant and evaporated to dryness. The residue was resuspended in water, containing 0.1% acetic acid. An aliquot of the extract was filtered and analysed using LC/MS/MS (Acquity UPLC BEH C18 column, water containing 0.1% acetic acid/acetonitrile gradient, Ultima Pt). Standards of lycopsamine, heliotrine, heliotrine *N*-oxide, retrorsine, senecionine, echimidine, jacobine, jacobine *N*-oxide, trichodesmine—and at a later stage also lasiocarpine—were available. PAs for which no standards were available were quantified using standards of the above-mentioned PAs having a similar structure. During the analytical sessions, duplicate determinations were performed at regular intervals. Method recovery percentages were close to 100%. Therefore, the results were not corrected for recovery. The results of the duplicate determinations were within the expected variability, associated with possible inhomogeneity of samples and analytical methodology. In the blanks no PAs were found.

### 2.5. Statistical Issues

We assessed different exposure factors using univariate and multivariate analysis. Potential confounders were identified on an *a priori *basis and included in multivariate logistic regression analysis. A restricted analysis was also conducted which separately examined the different categories of controls. Secondarily, an analysis was conducted which compared exposure factors between cases and cohabiting controls in order to assess intrahousehold differences, if any. 

To assess food consumption, we first reviewed the frequency of consumption of each food item. Then, removing those items rarely eaten, we developed index scores for five different food groups, summing the number of times each food item was reportedly eaten weekly in summer and in winter, and totaling food items in each of five food groups. Components of each food group were as follows. Protein: meat (beef, lamb, and goat), liver, chicken, goat milk, cow milk, qurut, yogurt, egg, chickpeas, lentil, and beans; Dairy: goat milk, cow milk, egg, yogurt, and qurut; Pulse: peas, lentils, and beans; Fruit: apple, melon, and watermelon; and Vegetables: carrot, and tomato. The food group scores were then divided into ordinal categorical variables. Protein consumption was categorized into quartiles, while other food groups were categorized into binary variables, either above or below the median score or classified as “ever” or “never” consumed. Frequency of sole bread consumption was similarly summed across seasons and categorized by quartiles. In addition, an overall food consumption level was assessed using the sum of all index scores divided into quartiles.

Socioeconomic status was assessed using principle components analysis [15] with household assets, employment, and education of head of household serving as variables in the model to define socioeconomic quartiles.

## 3. Results

### 3.1. Epidemiological Assessment

In total, 67 residents of Gulran district met the case definition and were included in the study. Cases were identified from among 28,443 individuals surveyed, including 3,700 households, the district and provincial hospital, and affected clinics in Gulran district. Point prevalence was 0.24% [95%CI 0.18–0.3%]). Table 1 shows enrollment characteristics of cases and controls. Self-reported onset of symptoms was reported progressively from October 2007, with a peak of 14 cases in February 2008. All cases reported exposures in Gulran district and enhanced surveillance in surrounding districts revealed no additional cases. By local report, a small number of cases sought care in neighboring Iran and are not included here. 

One-hundred and ninety-nine controls met the inclusion criteria and were included in the study. Nineteen of these (9.5%) were from the households of cases, 99 (47.7%) were from the same village as cases, and 81 (40.7%) were from villages in unaffected areas. The study was designed as a frequency matched study for age group and sex so there were no differences in proportion of each of these variables between cases and community controls, although there was a nonsignificant difference in age group between household cases and controls (Table 1). 

Table 2 shows results from risk factor analysis. Those who frequently consumed bread as the sole item in a meal had higher odds of disease; (adjusted odds ratio (AOR) 35.8 [95%CI: 7.6–168.2]) and those who had relatively high protein consumption were at decreased risk of disease (AOR: 0.1 [95%CI: 0.02–0.9]). High rice consumption (suggestive of high carbohydrate diet) was associated with disease (AOR: 2.6 [95%CI: 1.1–5.9]). High fruit consumption was negatively associated (AOR: 0.2 [95%CI: 0.06–0.6]). The magnitude of the association with carbohydrate and fruit intake compared to that for bread and protein consumption suggests that these are minor contributory factors. Water source other than a spring (well, pump, or other source of water) was associated with disease. There was no clear trend in the association of socioeconomic status to odds of disease. Having *charmac* growing on the household's land was associated with disease (AOR: 8.5 [95%CI: 2.1–33.1]). The level of consumption of individual food stuffs was also examined individually, but no single item appeared to be associated with disease. Amongst these were *qurut* and other dairy products (goats and cows milk, yogurt). 

Controls (*n* = 19) who cohabited with cases (*n* = 19) were assessed using Mantel-Heintzel test for trend. Within households, frequent consumption of bread as the sole food item was associated with disease, controlling for protein level (MH-OR 2.7 [95%CI: 1.3–5.5]). By this analysis, none of age, sex, or other food intake levels were associated with disease on univariate analysis.

Liver function was also measured amongst cases and controls. Analysis shows that cases more frequently had elevated levels of aspartate aminotransferase (SGOT), alanine aminotransferase (SGPT), bilirubin, and mean (and geometric mean) levels differed between cases and controls (Table 3). Tests for hepatitis B antigen and hepatitis C were also conducted to be assessed as risk factors for disease, but difference in prevalence between cases (Hep B: 7.6%; Hep C: 1.5%) and controls (Hep B: 2.6%; Hep C: 1.0%) was not significant.

### 3.2. Laboratory Analysis for PAs

**Figure 1:**
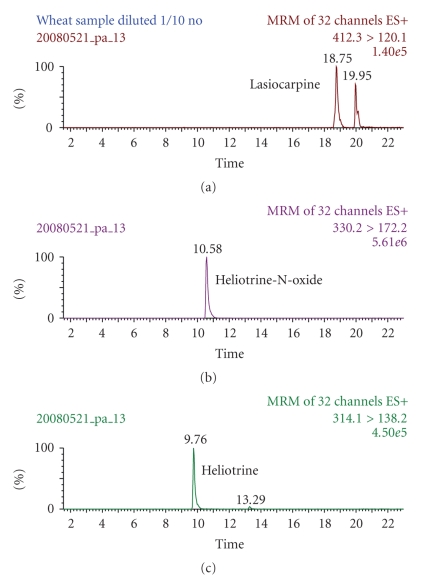
Chromatogram of wheat sample from a suspected area. The traces (mother > daughter ions) shown are those from the strongest transition of the two traces acquired for each PA.

During the survey samples of flour, grain, *qurut*, and plant matter were collected. Thirty-two samples of flour were collected from a convenience sample of 17 households and analysed for PA by LC/MS/MS. Median total concentration of PA in these flour samples was 4.0 mg/kg. Amongst samples taken from the houses of cases (*n* = 12), median concentration of total PA was 5.6 mg/kg (95%CI: 0.4–38.38), whereas amongst controls (*n* = 20) the median concentration was 2.7 mg/kg (95%CI: 0.4–4.0) (Wilcoxon rank-sum test: *z* = 1.6, *P* = .1). Heliotrine, lasiocarpine and heliotrine-N-oxide were detected in all flour samples with higher levels of heliotrine found in samples from the houses of cases (Wilcoxon rank-sum test: *z* = 2.0, *P* = .05) (Table 4). A chromatogram of a sample of wheat grain collected in a suspected area is given in Figure 1. Samples of stems and leaves (*n* = 2), roots (*n* = 1) and seeds (*n* = 1) of the *charmac* plant also contained PAs (Table 5).

PA concentration from the 32 flour samples was categorized into 3 groups on a logarithmic scale from 0-1 mg/kg, 1–10 mg/kg, and 10–100 mg/kg. PA levels in flour were higher amongst those who had charmac growing on their land (Fisher's exact, *P* < .001), those who had charmac growing amongst their wheat (Fisher's exact, *P* < .001), and those whose primary source of wheat was their own land (Fisher's exact, *P* = .007). There was a trend to suggest that samples collected from cases were more likely to be in the higher PA level (10–100 mg/kg) (OR = 9.5 [95%CI: 0.7–491.7], *P* = .053) although the sample size is small for this analysis.

*Qurut* and water samples were also analyzed as a possible source of PA in the diet. Water samples contained no PAs but they were found in the *qurut* samples (median concentration, 0.09 mg/kg [range: 0–0.6]). The ratios between heliotrine and heliotrine N-oxide in the *qurut* samples (derived from goats milk) were reversed compared to those in the flour samples. This can be expected because of metabolic action in the goat. Therefore, contamination of these samples with flour is unlikely. Large amounts of trichodesmine, a PA not found in the charmac or wheat samples, were also found.

## 4. Discussion

Identified cases were significantly more likely than controls to report frequent consumption of bread and samples of flour were found to be contaminated with high levels of PA. The principle source of PA appears to be wheat flour contaminated with the seeds of the *Heliotropium* plant which commonly grows with wheat in this area. A secondary, though minority, source is likely to be *qurut* (whey) from the milk of goats which have ingested plants containing PAs while grazing. Consumption of a high ratio of bread to other food-item consumption was strongly associated with disease, while a diet high in protein appeared to be protective. This association was also seen within households, using a matched analysis which may result from different behavioral factors related to diet within the household. It seems likely, therefore, that a combination of prolonged, regular exposure to contaminated wheat in the presence of a low-protein diet is chiefly responsible for clinical presentation. Those with a more varied diet were also less likely to be cases.

The primary analysis is partly based on an index measure of self-reported food intake over two seasons which recorded the number of times an individual food item was eaten per week. The sum of the frequencies provided an intake score for each food group. This provided an indirect measure of food intake which may be subject to recall and other biases of self-reported dietary intake assessments [16]. 

Numerous PAs have been identified which have been shown to be hepatotoxic in human and animal models. The mechanism of action of PA is well established [17]. PAs are not toxic* per *se but require metabolic oxidation by hepatic multifunction oxidases to pyrrole derivatives, which then undergo hydrolysis to strongly alkylating metabolites [17]. In general, the diester PAs are more toxic than the monoesters; thus, the diester lasiocarpine is more hepatotoxic than the monoester, heliotrine.

Some PAs in the *qurut* are not present in *charmac* (i.e., trichodesmine), suggesting that the animals are grazing on other plants that contain PA and indicating potentially that *charmac* is not the only plant responsible for the human exposure to PAs. Although the amount of PAs in the *qurut* is in general much less than in wheat samples tested, this route of exposure may also be a contributory factor.

The amount of PA contained in the* charmac* is consistent with other* Heliotropium* species. A total alkaloid content of 0.76% for whole plant and 1.7% for seed has been found in other heliotropic plants, and thus a level of 1.9% for the Afghanistan samples is not unreasonable. Estimated dose and duration of exposure to PA required to produce liver damage in humans has been previously noted [18]. For heliotrine, an intake of 4–10 mg/kg per day for 3–7 weeks could lead to necrosis and VOD. The WHO has indicated a lowest intake rate of 1 mg total PAs per day (for a 70 kg adult) [4] with German regulations for herbal remedies establish a maximum oral intake of 1 microgram per day [19]. The high PA levels in the Afghanistan samples could certainly result in toxic exposure. 

Sustained action is required to ensure the long-term safety of food. Amongst these is to identify appropriate technologies for elimination of the contamination, development of in-country capacity to assess PA content in samples, and implementation of routine testing in historically affected areas. 

Global food shortages, changes in local ecology due to climate change, and ongoing lack of development in agricultural sectors make food safety an important public health issue. As food prices increase, it is likely that the proportion of the rural poor who rely on subsistence farming and locally traded produce will increase. If adequate food-safety practices are not employed at this level of society, incidents such as this outbreak are likely to become more common.

## Figures and Tables

**Table 1:** Demographic characteristics of cases and controls.

	Case	All Controls	Household Controls	Villages controls	District controls
*N*	67	199	19	99	81
Sex (% male)	39/67 (58.2%)	117/199 (58.8%)	7/19 (36.8%)	62/99 (62.6%)	48/81 (59.3%)
Mean Age, years (SD)	24.6 (16.0)	24.7 (16.1)	21.9 (11.6)	24.8 (16.5)	25.3 (16.7)
Age group					
0–10	14/67 (20.9)	40/199 (20.1)	2/19 (10.5)	20/99 (20.2)	18/81 (22.2)
11–20	20/67 (29.9)	62/199 (31.2)	8/19 (42.1)	34/99 (34.3)	20/81 (24.7)
21–30	13/67 (19.0)	38/199 (19.1)	6/19 (31.6)	14/99 (14.1)	18/81 (22.2)
>30	20/67 (29.9)	59/199 (29.6)	3/19 (15.8)	31/99 (31.3)	25/81 (30.9)
Median number people per household (inter-quartile range)	7 (5–9)	9 (7–13)	9 (8–18)	10 (7–15)	9 (6–12)

**Table 2:** Univariate and multivariate analysis of potential exposure factors for hepatic veno-occlusive disease in Gulran District, Afghanistan, 2008. Odds ratios presented with 95% confidence intervals in parentheses.

Variable	Univariate Odd Ratio	Multivariate Odd Ratio
Socioeconomic quartile		
(Poorest)	1	1
2	0.3 (0.1–0.7)	0.4 (0.1–1.1)
3	0.2 (0.06–0.4)	0.3 (0.1–1.0)
Least poor	0.3 (0.2–0.7)	0.8 (0.3–2.1)

Wheat source (not home grown)	2.3 (1.4–3.9)	1.8 (0.9–3.9)
Remove charmac seeds	0.5 (0.3–0.9)	Removed due to missing data
Charmac Grows on land	5.0 (1.7–14.5)	8.5 (2.1–33.1)

Bread consumed (alone)		
Infrequent	1	1
1	2.0 (0.7–5.6)	6.3 (1.6–24.8)
2	1.6 (0.6–4.2)	3.6 (1.0–13.3)
Frequent—3	10.3 (3.3–32.1)	35.8 (7.6–168.2)

Protein Level		
Low	1	1
1	0.4 (0.2–0.8)	0.6 (0.2–2.0)
High-2	0.2 (0.1–0.5)	0.1 (0.02–0.9)
		
High Dairy Level	0.5 (0.3–0.9)	1.2 (0.4–3.7)
		
High Pulse Level	0.5 (0.3–0.9)	0.7 (0.3–1.9)
		
High Fruit Level	0.3 (0.2–0.7)	0.2 (0.06–0.6)
		
High Rice Level	0.9 (0.5–1.7)	2.6 (1.1–5.9)
		
High Food Level	0.4 (0.2–0.7)	2.1 (0.6–6.8)

**Table 3:** Comparison of liver function between cases and controls, Gulran District, Afghanistan, 2008.

	*n*/*N* (%) with elevated levels		Mean/Geometric mean	
Test	Cases	Controls	Cases	Controls
SGOT (IU/l)^1, 2^	32/66 (48.5)	25/194 (12.9)	36.6	27.9
SGPT(IU/l)^1, 2^	30/66 (45.5)	25/194 (12.9)	42.3	33.1
Alk Phos (IU/l)^2^	2/66 (3.0)	2/194 (1.0)	213.2	182.9
Bilirubin (mmol/l)^1, 2^	29/66 (44.0)	21/194 (10.8)	0.8	0.5
				
Hep B Antigen	5/66 (7.6)	5/194 (2.6)	—	—
Hep C Antigen	1/66 (1.5)	2/194 (1.0)	—	—

^1^Chi Sq, *P* < .001.

^2^Students*T*-test (log data if skewed): *P* < .001.

**Table 4:** PA content of thirty-two flour samples collected from affected villages, Gulran District, Afghanistan, 2008.

	Flour Samples median, (mg/kg)	
	Cases *n* = 12	Controls *n* = 20

Heliotrine^a^	0.16	0.07
Heliotrine-N-oxide	5.4	2.6
Lasiocarpine^b^	0.045	0.025
(quantified against echemidine)		

TOTAL PA	5.6	2.7

Mann-Whitney rank-sum test between cases and controls for flour samples:

^a:^ *z * = 2.0, *P* = .050

^b:^ *z * = 1.9, *P* = .057.

**Table 5:** PA content of four specimens of charmac plant matter collected from affected villages, Gulran District, Afghanistan, 2008.

	Plant Matter Sample (mg/g)			
	Stems and leafs	Stems and leafs	Fresh roots	Seeds

Heliotrine	343	112	67	262
Lasiocarpine	459	252	81	698
(quantified against senecionine)				
Jacobine	158	98	60	95
(quantified against senecionine-N-oxide)				

TOTAL PA	960	462	208	1055
